# Correlation between Histopathological and FT-Raman Spectroscopy Analysis of the Liver of Swiss Mice Infected with *Paracoccidioides brasiliensis*

**DOI:** 10.1371/journal.pone.0106256

**Published:** 2014-09-02

**Authors:** Elaine Sciuniti Benites Mansano, Gutierrez Rodrigues de Morais, Edilaine Martins Moratto, Francielle Sato, Antonio Medina Neto, Terezinha Ines Estivalet Svidzinski, Mauro Luciano Baesso, Luzmarina Hernandes

**Affiliations:** 1 Department of Morphological Sciences, Universidade Estadual de Maringá, Maringá, Paraná, Brazil; 2 Department of Physics, Universidade Estadual de Maringá, Maringá, Paraná, Brazil; 3 Department of Clinical Analysis and Biomedicine, Universidade Estadual de Maringá, Maringá, Paraná, Brazil; University of Minnesota, United States of America

## Abstract

Paracoccidioidomycosis is the most important systemic mycosis in Latin America. The main entrance of the fungus is the airway. It primarily occurs in the lung, but in its disseminated form may affect any organ. The liver is one of the organs afflicted by this disease and its homeostasis may be impaired. The aim of the present study was to evaluate the evolution of paracoccidioidomycosis in the liver of Swiss mice and correlate morphological factors with the expression of gp43 and with physicochemical analysis via FT-Raman of the infected organ. According to colony forming unit (CFU) and granuloma counting, the first and second weeks were the periods when infection was most severe. Tissue response was characterized by the development of organized granulomas and widespread infection, with yeasts located within the macrophages and isolated hepatocytes. The gp43 molecule was distributed throughout the hepatic parenchyma, and immunostaining was constant in all observed periods. The main physicochemical changes of the infected liver were observed in the spectral ranges between 1700–1530 cm^−1^ and 1370 – 1290 cm^−1^, a peak shifting center attributed to phenylalanine and area variation of -CH_2_ and -CH_3_ compounds associated to collagen, respectively. Over time, there was a direct proportional relationship between the number of CFUs, the number of granulomas and the physicochemical changes in the liver of mice infected with *Paracoccidioides brasiliensis*. The expression of gp43 was similar in all observed periods.

## Introduction

Paracoccidioidomycosis (PCM) caused by *Paracoccidioides spp*. is endemic in humid tropical and subtropical areas of Latin America where it is considered one of the most important mycoses and, among chronic infectious and parasitic diseases, represents the eighth leading cause of death from infectious disease. It has the highest mortality rate among systemic mycoses [1]–[3]. It is granulomatous in character and grows insidiously. It is of considerable medical importance, as it affects people in their productive phase, making them unable to work and resulting in sequelae [4], [5].

*Paracoccidioides brasiliensis* (Pb) is the main agent of PCM. It is a thermally dimorphic fungus: it appears as mycelium at 25°C and as yeast at 36°C [6]. In yeast form it can spread through the body of the host and parasite deeper tissues [7], [8]. The occurrence of yeasts within polymorphonuclears, monocytes and macrophages shows that this strain can survive within these cells [9].The principal antigenic component of Pb is a 43 kDa glycoprotein known as gp43 [10] which is synthesized and transported to the cell wall and then secreted to the medium [11]. There is evidence that gp43 participates in adhesion, invasion and pathogenesis of the fungi [12], inhibits the fungicidal activity of macrophages, and contributes to the development of infection in susceptible hosts [13]. Additionally, gp43 is the main known antigen for the diagnosis and prognosis of PCM [14]. Diagnosis of natural PCM is currently based on the appearance of the agent in tissue. However, this diagnostic process is limited, and serological techniques have been proposed as an alternative [15], [16].

A new perspective for the diagnosis of PCM is via Fourier Transform Raman Spectroscopy (FT-Raman). This technique allows the characterization of molecular components and detects, by the Raman bands, structural deformations resulting from infection [17]. In fact, infrared methods can accelerate the diagnosis of diseases and represent a breakthrough in experimental investigations of PCM [18], [19].

The main organ affected by PCM is the lung, but can often affect other sites such as bone, skin, mucosa and lymphoid organs [2], [3], [20]. The liver can also be affected by this disease, and some studies show that liver enzymes are altered [21], [22] indicating that the infection can disrupt the homeostasis of this organ. However, there are no studies of the liver of animals infected with Pb using FT-Raman spectroscopy.

The aim of this study was to evaluate the morphological evolution of paracoccidioidomycosis in the liver of *Swiss* mice and to correlate morphological changes with the expression of gp43 and with physicochemical analysis of the infected organ using FT-Raman spectroscopy.

## Materials and Methods

### Fungal Isolate

A sample of *Paracoccidioides brasiliensis* (Pb 18 isolate) was obtained from the Fungal Culture Collection of the Department of Immunology of the Universidade Federal de São Paulo (UNIFESP) and stored in Fava Neto culture medium at 35°C in the Medical Mycology laboratory of the Universidade Estadual de Maringá (UEM).

### Animal Experimentation

All procedures involving animals were approved by the Ethics Committee in Animal Experimentation (CEEA) of the Universidade Estadual de Maringá, under register number 116/2010.

A total of 32 *Swiss* male mice, weighing 30 g and aged between 4 and 5 weeks, were used. The animals were transferred from the Central Animal Laboratory to the Laboratory for Paracoccidioidomycosis Experimentation of the Department of Basic Health Sciences. The animals were kept under controlled environmental conditions, with a temperature of 23–24°C, and a light/dark cycle of 12 hours with free access to water and food.

### Infection of animals with *Paracoccidioides brasiliensis* fungal isolate

The mice were divided into four groups of eight, six of which were infected and two were used as control. The animals were anesthetized by intramuscular injection of a solution containing 100 mg/kg of ketamine hydrochloride (Park, Davis & Company, Berlin, Germany) and 10 mg/kg xylazine (Bayer, Brazil) [23]. After the anesthesia the mice were infected, via the lateral tail vein, with 0.1 ml of a fungal suspension containing 2×10^6^ yeast cells of Pb per milliliter of colony forming units (CFU/ml). Control animals received 0.1 ml of sodium phosphate buffer (PBS), pH 7.4, in the same manner.

### Death of animals

Animals from both infected and control groups were killed 1, 2, 4 and 8 weeks after inoculation, with an overdose (40 mg/kg) of sodium thiopental (Crystal Pharma, Minas Gerais, Brazil) in the lateral tail vein. The liver was removed and the right lobe was isolated and fixed in 4% paraformaldehyde solution for 24 hours and processed for paraffin embedding. Five mm thick semi-serial cuts were made. Fragments of the left lobe were weighed and placed in sterile test tubes with 1 ml of PBS then macerated with a pistil to obtain a homogenous suspension. This suspension was used for counting the CFUs. The remainder of the left lobe was used for spectroscopic study by FT-Raman.

### Identification of CFUs/g of liver tissue

A volume of 300 µl of the infected liver suspension was placed in Petri dishes containing brain heart infusion (BHI) agar medium (Difco Laboratories, Detroit, Michigan) supplemented with 5% fetal bovine serum and growth factors [24]. The plates were incubated at 35°C for 15 days. CFUs/g counting was performed on the seventh and fifteenth days of incubation and the log_10_ of the CFUs/g was calculated.

### Histological and immunohistochemical study

Histological sections of liver were stained with: (a) hematoxylin and eosin (H&E) and (b) periodic acid-Schiff (PAS) for histopathological evaluation; (c) sirius red by picrosirius technique to assess the presence of fibrosis; (d) impregnated by silver-methenamine in accordance with Gomori-Grocott and counter-stained with light green or H&E [25], [26] for locating the yeast in the hepatic parenchyma; and (e) immunostained with anti-gp43 antibody to detect expression of the glycoprotein gp43.

Expression of gp43 in the liver was detected by avidin-biotin peroxidase immunohistochemical staining method using polyclonal primary antibody, anti-gp43 obtained in rabbits, and diluted in a concentration of 1∶50, and revealed using a immunohistochemical commercial kit (Histostain-Plus kits, Invitrogen 2^nd^ Generation, LAB-AS Detection System, Camarilo, CA).

Slides were observed and photographed using a trinocular light microscope (Nikon Eclipse 80i), with a camera (Nikon DSFi1C) coupled to a computer using Nis Element software (Shinjuku, Japan).

### Granuloma Counting

Granuloma counting was performed in histological sections stained with H&E. Granulomas present were counted in four histological/animal sections (24 sections/week of infection).

A histological section of the liver was outlined in order to create a chart that plotted the positions of the granulomas found in all of the sections analyzed, thereby avoiding repeated counts of the same granuloma. The counts were carried out using a 20× objective on a microscope (Nikon Eclipse 80i).

To determine the approximate area in which the counting was carried out, the image of histological section of the liver of each animal was captured and its area determined using an image analysis program (Image Pro Plus, version 4.5). A mean area of (15.25±0.85) mm^2^ was calculated and used as a standard for the area in which the granulomas were counted. The numbers of granulomas were therefore expressed as number of granulomas/15.25 mm^2^.

### Fourier Transform Raman Spectroscopy (FT-Raman)

The analysis was performed on a FT-IR spectrometer coupled to a Raman module (Vertex 70 – RAM II module, Bruker, Germany), equipped with a Nd-YAG laser excitation source at 1064 nm and a germanium detector cooled to liquid nitrogen. The power used was 70 mW. Liver samples with a surface area of approximately 1 cm^2^ were analyzed in the spectral range between 3300 – 1200 cm^−1^. The spectra obtained for each sample represented an average of 400 scans with spectral resolution of 4 cm^−1^. The analyzed areas for all samples were the same since the adopted diameter for the excitation laser spot was around 4 mm.

### Statistical Analysis

Variance analysis (ANOVA) was performed to assess the number of CFUs/g and of granulomas, using Graph Pad Prism 3.0 software (Graph Pad Software, Inc., La Jolla, CA, USA). The Tukey post-test was used with a 95% confidence level.

## Results

### 1. Counting of Colony Forming Units - CFUs

**Figure 1:**
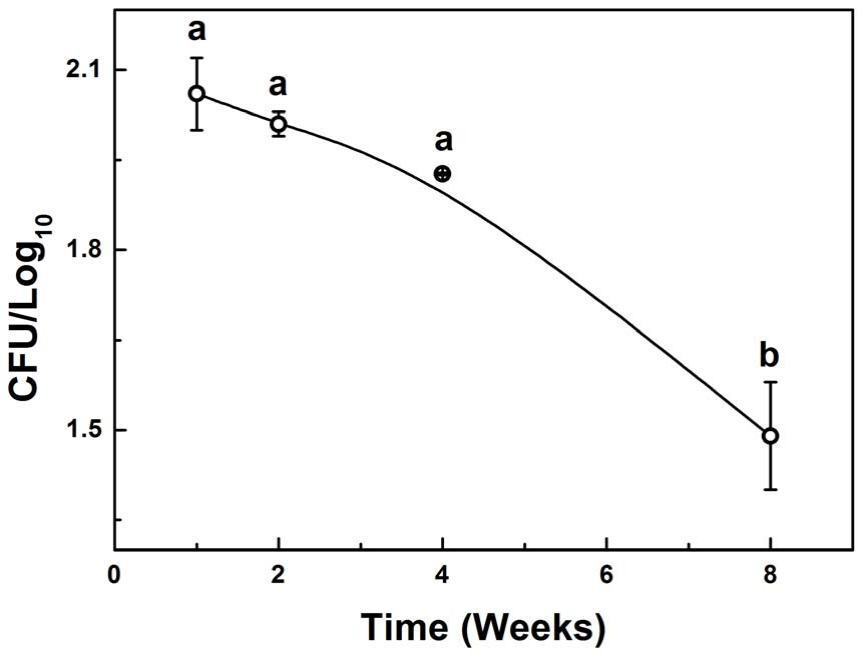
Number of Colony Forming Units (CFUs)/g in the liver of mice infected with *Paracoccidioides brasiliensis*. Results represent mean ± EPM and were expressed as Log_10_; n = 6. Different letters represent P<0.001. ANOVA, Tukey post-test.

The number of CFUs of the fungus found in the liver of the animals decreased after the first week, with a significant reduction between the 4^th^ and 8^th^ weeks of infection (Figure 1).

### 2. Granuloma Counting

**Figure 2:**
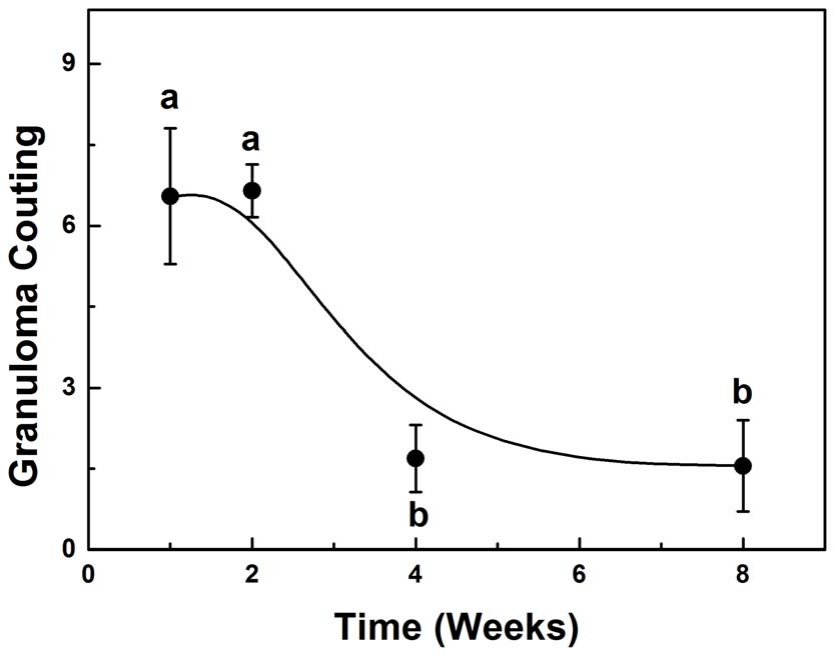
Number of granulomas in the liver of mice infected with *Paracoccidioides brasiliensis* as a function of time infection. Results expressed as the mean ± EPM. Different letters represent P<0.001. ANOVA, Tukey post-test.

The granuloma counting results are shown in Figure 2. The granulomatous lesions decreased between the second and fourth weeks but remained constant in the other periods.

### 3. Histopathological Study

In the first weeks of observation, the formation of granulomas was one of *t*he most obvious changes in the hepatic parenchyma of infected mice.

**Figure 3:**
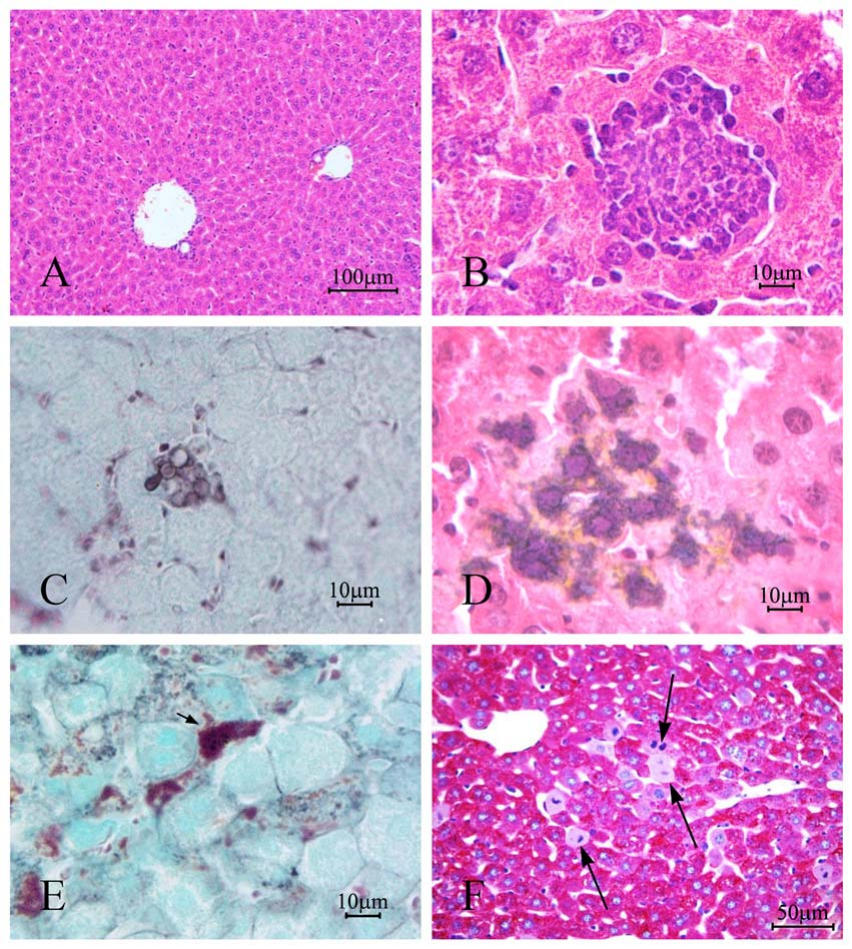
Photomicrograph of: (A) liver of mice control, non-infected and (B - F) after one week of infection with *Paracoccidiodes brasiliensis*. In (B) yeasts were not observed in the stained granulomas with H&E, though sprouting and varied shapes were indentified with (C) Gomori-Grocott staining; in (D) and (E) note the presence of fungical fragments impregnated with silver within hepatocytes and macrophages (arrow); in (F) large number of mitosis (arrow) were observed in the hepatic parenquima. Stain: H & E (A and B); Gomori-Grocott counter-stained with green light (C and E) or H & E (D); Periodic acid-Schiff (PAS)(F).

In the first week (Figure 3) the granulomatous lesions occurred predominantly in the periphery of the organ. They appeared as well-defined structures consisting of aggregates of histiocytes, macrophages, plasma cells, lymphocytes and neutrophils. In this period, the yeasts were evident with their characteristic morphology within the granulomas, and could be viewed only with Grocott-Gomori staining. Eventually, isolated macrophages and hepatocytes revealed impregnation by silver. Other histological changes such as the presence of mitosis and eosinophilic cytoplasm of the hepatocytes were observed in the first week of infection, the period in which the largest number of CFU (2.05 CFU/log_10_) occurred (Figure 1).

**Figure 4:**
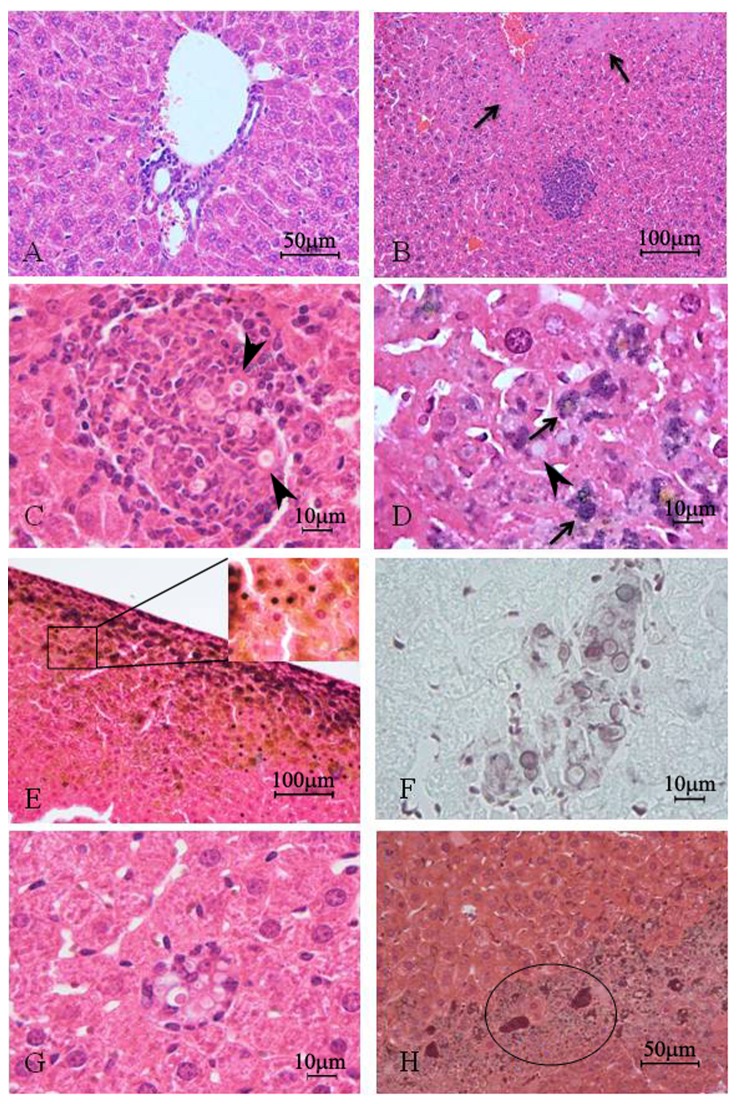
Photomicrograph of: (A) liver of mice control, non-infected and (B-H) after two weeks of infection with *Paracoccidiodes brasiliensis*. The hepatic parenchyma showed more developed granulomas (B and C) and more eosinophic areas (arrows) (B); the yeast were visualized inside the H&E stained granulomas (C); the hepatic parenchyma showed intracytoplasmic fungal fragments (arrows) and karyolysis (D); the infection was more evident in the organ periphery where several pyknotic nuclei were identified (E); the granulomas showed high number of yeast (F); in this period large macrophages with yeast inside (circled area) (G) and large necrotic areas (H) were observed. Stain: H&E (A, B, C and G); Gomori-Grocott counter-stained with H&E (D, E and H) or green light (F).

In the second week (Figure 4) liver disease presented two morphological patterns: (1) areas with organized granulomas well defined, containing yeast, interspersed with areas of normal parenchyma, and (2) other areas with diffuse infection without clear limits where the hepatic parenchyma had necrotic areas with cytoplasm acidophilic, pyknosis and nuclear chromatolysis. In these regions, the yeasts appeared isolated, intact or fragmented within hepatocytes. Fragmented yeasts impregnated by silver were identified within macrophages. A total of 100% of the samples revealed yeast, with or without budding.

**Figure 5:**
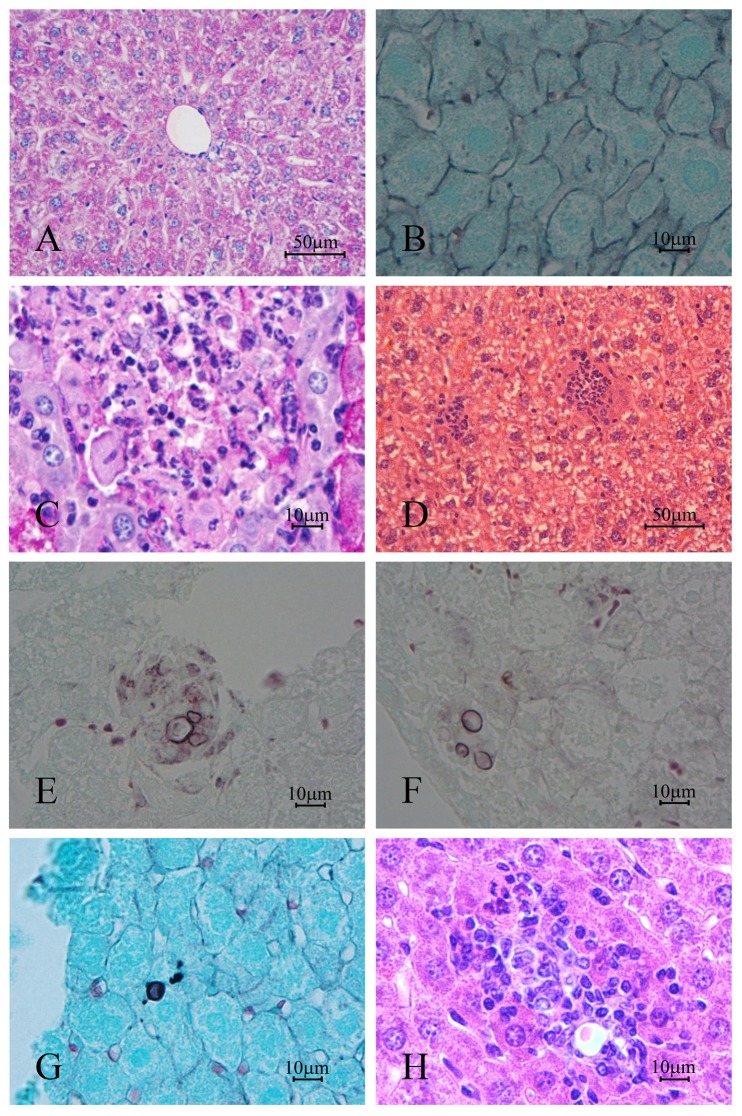
Photomicrograph of: (A and B) liver of mice control, non-infected and (C - F) after four (G and H) and eight weeks of infection with *Paracoccidiodes brasiliensis*. Organized granulomas (C) with a fewest or without yeasts (C-F) were observed on the fourth week. On the eighth week yeasts were still observed. Stain: Periodic acid-Schiff (PAS) (A and C); Gomori-Grocott counter-stained with green light (B and E-F) or H&E (D and H).

Sections after four and eight weeks of infection (Figure 5) showed morphological features similar to those described for previous weeks, but in reduced proportions (fewer granulomas, reduced extent of lesions and fewer CFUs). At the same time the number of animals with granulomatous lesions also varied (77% in the fourth week and 50% in the eighth week). The eighth week had the lowest number of CFUs.

**Figure 6:**
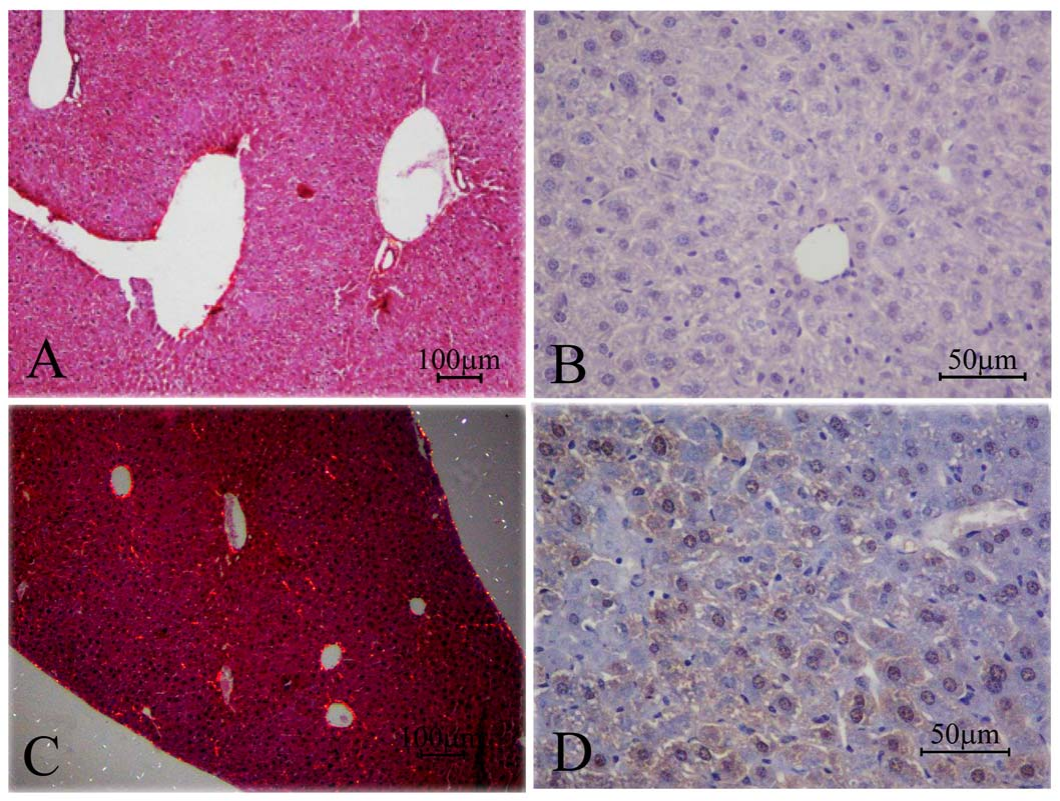
Photomicrograph of: (A and B) liver of mice control, non-infected and (C and D) after eight weeks of infection with *Paracoccidiodes brasiliensis*. Note an increase in the number of birefringent collagen fibers (C). Immunhistochemistry using anti- gp43 antibody showed diffuse cytoplasmic and nuclear staining in the liver parenchyma (D). Stain: Picrosirius technique (A and C); Avidin-biotin peroxidase immunohistochemical using anti-gp43 antibody (B and D).

There was an increase over time in the amount of collagen fibers concentrated close to the sinusoidal capillaries between the hepatic cords (Figure 6).

Anti-gp43 expression occurred in both regions; nuclear and cytoplasmic of the hepatocytes presenting an imunostaining diffuse pattern. Yeast with marking was rarely seen. Instead, large areas were immunostained throughout the parenchyma. The staining was similar in all the observed periods (Figure 6).

### 4. Physicochemical analysis by Fourier Transform Raman Spectroscopy

**Figure 7:**
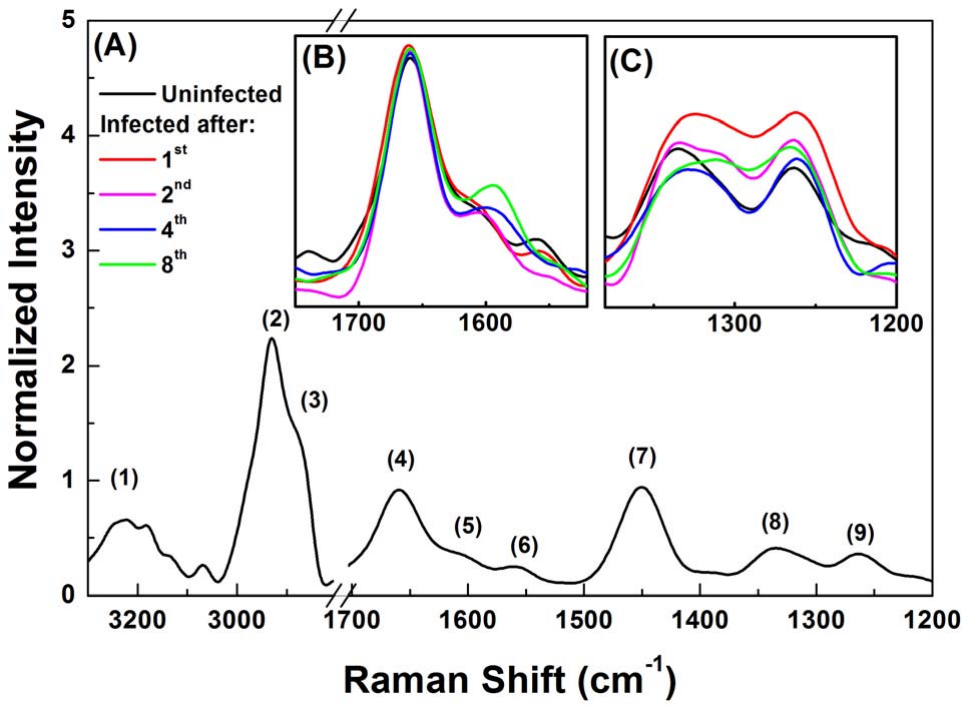
Raman spectrum from liver of mice (A) control, non-infected and (B and C) after one, two, four and eight weeks of infection with *Paracoccidioides brasiliensis*. The insets show the spectral range between 1750-1520 cm^−1^ (B) and 1380-1200 cm^−1^ (C) where the spectral difference compared non-infected with infected tissues were observed.

The results show that there are spectral differences in the liver of mice infected with Pb compared to those of normal mice (Figure 7).

**Figure 8:**
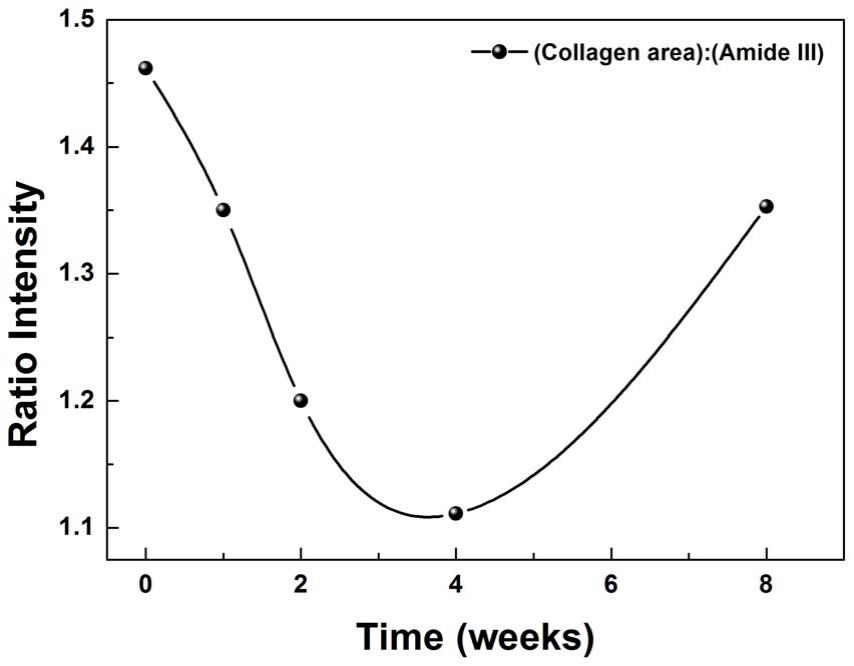
Comparison of area ratio (1340 cm^−1^/1260 cm^−1^) between mice non-infected liver tissue (t = 0) and after one, two, four and eight weeks of *Paracoccidioides brasiliensis* infection.

The functional groups associated to each numbered peeks and their respective attributions are shown in Table 1 and Figure 7 (A). All spectra were subjected to baseline correction and normalized with regard to the peak of the amide I (1660 cm^−1^). Figures 7 (B) and 7 (C) shows the regions in which Gaussian fitting, for obtaining the position and area of the characteristic peaks of infected and non-infected tissue, were performed. Figure 7 (C) shows the spectra between 1370-1290 cm^-1^, associated with the deformation of -CH_2_ and -CH_3_, and in this region there was a variation in the area with increased time of infection. The functional groups, -CH_2_ and -CH_3_ are usually correlated to the presence of collagen fibers in the tissues [27]–[29]. This variation was investigated using the ratio between the area of the collagen and amide III regions (1260 cm^−1^), a nearby region to the collagen, which remains stable during the studied time of infection. The results are presented in Figure 8 in which the behavior of this ratio comparing the control (t = 0) with the infected liver after one, two, four and eight weeks, showing a decay of the collagen area in the first two weeks, followed by an increase after the fourth week of infection.

**Table 1:** Non-infected mice liver Raman bands and its assignments.

Peak N°	Raman shift (cm^−1^)	Band assignments
1	3220	v(N-H) (Protein related) [57]
2	2927	v_as_(C-H) (Lipids and protein related) [27]
3	2874	v_s_(C-H) (Lipids and protein related) [27]
4	1660	Amide I (Protein related) [29]
5	1615	Tyrosine (amino acid) [28]
6	1557	δ_as_(NH_3_^+^) (Red cell blood contribution) [27], [58]
7	1450	δ(CH_2_) (Lipids and protein related) [27]
8	1340	δ(CH_2_) and δ (CH_3_) (Collagen)[27], [29]
9	1260	Amide III [27], [28]

v: stretching vibration; v_as_: asymmetric stretching vibration; v_s_: symmetric stretching vibration; δ_as_: asymmetric bending vibration; δ_s_: symmetric bending vibration.

**Figure 9:**
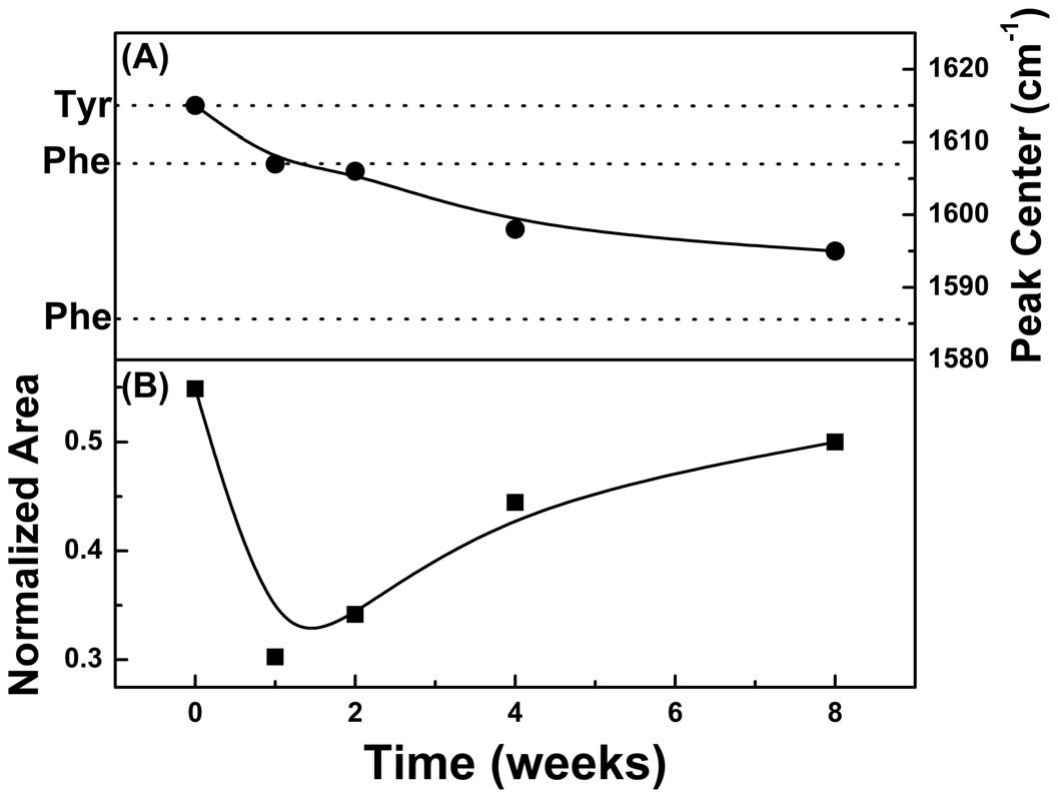
(A) Shifting of 1615 cm^−1^ peak position observed in non-infected liver (t = 0) toward low frequencies as a function of time infection. (B) Behavior of amino acids band area (1710-1510 cm^−1^) as a function of time infection.

Figure 9 (A) shows the peak displacement of 1615 cm^−1^ identified in the spectrum of the control liver toward lower frequencies as a function of infection time, reaching a value of 1595 cm^−1^ in the eighth week. This region can be related to amino acids in the liver itself or in the blood. The peak at 1615 cm^−1^ is attributed to tyrosine [27], [28] and at 1585 cm^−1^ to phenylalanine [27], [28]. Figure 9 (B) shows the behavior of the total area related to these amino acids (1710-1510 cm^−1^) as a function infection time.

There is a decrease in the amino acids content in the first week of infection compared to control tissue and a tendency of increasing up to the eighty week. From the second week on there is a tendency to increase the total area of the amino acids. However it should be considered that the greatest contribution in the control liver is due to the presence of tyrosine, and for the infected tissue it is from the phenylalanine.

## Discussion

In this study, FT-Raman technique and histological staining were used to assess the behavior of the liver infected with Pb from a morphological and physicochemical perspective. The expression over time of the major antigen produced by Pb, gp43, was assessed.

In natural PCM the liver is not the preferred organ, but it is affected from the pulmonary focus after fungal spreads via lymphatic and hematogenous route [30], [31], [2]. In comparison with studies of lungs, there are few clinical and experimental studies describing the involvement of the liver. Hepatosplenomegaly, jaundice and fibrosis are some of the described pathological alterations in patients infected with Pb [32]–[34]. However, it is important to mention that in the acute form of the PCM, that affects mainly children's and teenagers, the liver is between the most affected organ [35], [36].

In this study the intravenous route of infection of the Pb strain, one of the most virulent forms of Pb, seemed to promote the spread of the fungus and its installation in the liver. The infection was confirmed by CFU analysis, where fungal growth was evaluated.

Histopathological study using serial sections stained using different techniques proved, from different perspectives, the host-parasite relationship and the changes in the liver morphology. Hematoxylin and eosin staining and PAS technique identified granulomas containing yeast forms of Pb with clear halo and central acidophilic regions. Silver impregnation showed yeasts within the inflammatory infiltrate [37] and in the parenchyma of the liver.

In the liver stained in accordance with Gomori-Grocott, the structures impregnated by silver were located internalized within cells that were predominantly at the periphery of the liver, near the visceral peritoneum. These cells were identified as macrophages, because of their irregular shape and variable position, which suggests that these cells engulfed the yeasts to kill them, or that there were yeasts resident in these macrophages. Many hepatocytes also had small intracytoplasmic structures impregnated with silver, suggestive of internalized micro yeasts.

The method by which Pb can spread to organs is not fully understood. One way may be by migration of infected macrophages or dendritic cells through the lymph system [38], [39]. Yeasts have been found intracellularly in polymorphonuclear cells and in monocytes and macrophages, suggesting that the fungus is able to survive within these cells [9], [40], and can therefore remain hidden into the immune defenses of the host. It does not expose its antigen on the surface cell, escaping from professional phagocytes, facilitating therefore its dissemination [41].

In the present study, the number of fungi in the lesions decreased in the later periods of infection, suggesting that the granulomas formed in the first weeks represented an initial defense mechanism against Pb, affecting the multiplication and spread of yeast. It is possible that the more compact architecture of the liver, with few open spaces, may have hindered the establishment and spread of the fungus throughout the parenchyma, compared with the lung [37], which has ample space.

Moreover, it is possible that the mice developed a hyperergic form of the disease with cellular type immune response forming well-organized granulomas with few fungi, suggesting that these animals have high immunological resistance. Other authors have shown that *Swiss* mice are relatively resistant to Pb infection [42]–[44].

The formation of granuloma in experimental models and in humans depends on factors such as susceptibility or resistance to infection [45]. On the other hand, the disease development depends on the fungus virulence and antigenic composition, on the environmental conditions and the immune response of the host [46].

In a previous study using the same kind of murine species, the evolution of granulomas in the lungs [37] was morphologically evaluated over 1, 2, 4 and 8 weeks after infection by Pb. Over time, the pulmonary granulomas became more numerous, larger and more organized, with increasing amounts of collagen fibers and reticulars [37]. Contrastingly, in the liver, the number of granulomas was greater in the first two weeks of infection and declined significantly by the eighth week. Furthermore, granulomas became smaller over time and did not develop significant fibrosis.

For the first time, expression of gp43 was evaluated by immunohistochemical staining in the livers of infected animals. Glycoprotein gp43 is the main antigenic component used for the diagnosis of PCM [10]. The immunostaining of gp43 in the liver was predominantly diffuse, with cytoplasmic and nuclear expression and in some yeast forms intact and fragmented. The staining intensity remained similar throughout the experimental period.

The gp43 may be synthesized and stored in dense vesicles that appear to migrate to the outer edge of the fungal cell wall, and are excreted extracellularly. Gp43 was detected in the cytoplasm of the macrophages in the fungal cell wall, in the host cells cytoplasm as well as in a dispersed manner [41].

The present study used a physical chemical evaluation which may represent a new alternative for diagnosis of PCM, namely FT-Raman spectroscopy. Raman spectroscopy is a method that, combined with histopathological analysis, can aid diagnosis, and has been most commonly employed in cancerous cells and tissue [47]–[50]. It provides detailed information of the biomolecular composition of tissues, allowing normal tissues to be distinguished from diseased tissues [17]. There are no studies in existing literature of spectral analysis of the livers of *Swiss* mice infected with Pb.

Since liver is not the target organ of Pb, few studies have linked infection by this microorganism with hepatic damage. The analysis via FT-Raman suggested that the infection caused structural and functional changes in the organ. Figure 8 shows a change in the spectral region related to the protein collagen [27], [29]. The detected decrease of the collagen ratio, after the first week of infection, could be related to the Pb capacity to degrade the extracellular matrix, to adhere and to invade the host tissues. This process involves the interaction of the fungus with extracellular matrix proteins (ECM), such as enolase and adhesins. Adhesins are adhesion proteins in the Pb, which determine the binding capacity of this microorganism to matrix components such as collagen type I and IV, fibronectin, fibrinogen, and laminin [51], and also determine tropism by a cell or tissue [52], [53]. In turn, the enolase acts as a plasminogen receptor with proteolytic capacity, contributing to the degradation of the ECM and the establishment of the fungus in the host [7] body.

On the other hand, the increase in the collagen ratio from the fourth to the eighth week can be related to the chronicity of the infection. In the lung, Pb target organ, the PCM evolved into fibrosis, which is characterized by matrix deposition and collagen accumulation [35]. In the liver, there was the development of a typical case of fibrosis, but a larger amount of collagen fibers, stained with Sirius red, was observed with the progression of the infection. Fibrosis is an intrinsic mechanism to stop the evolution of the microorganism, allowing the establishment of an apparent balance between the host and the parasite, as the fungus cannot be eradicated, since the treatments only decrease the amount of fungi in the body maintaining the risk of late reactivation [2], [3]. In pediatric patients, the hepatic involvement is common and is related to a worse prognosis and a higher number of deaths. In these patients the amount of fungi present in the liver was associated with the intensity of fibrosis [36].

From a functional point of view, the infection seems to have influenced the amino acids metabolism. In the non-infected organs, no physicochemical changes were detected. In these animals a characteristic band of tyrosine amino acid has been identified (1615 cm^−1^). In Figure 9 (A), in the infected organ, there was a detection peak displacement from 1615 cm^−1^ to 1595 cm^−1^, related to tyrosine and phenylalanine, respectively, [28]. In the specific case of the conversion of phenylalanine to tyrosine, by phenylalanine hydroxylase, there is the incorporation of -OH radicals to the aromatic ring of phenylalanine, causing a rearrangement of loads in the ring, and consequently a peak displacement.

Figure 9 (B) shows the total area of the analyzed amino acid band, including tyrosine and phenylalanine (1710-1510 cm^−1^). The variation of the area may be related to the Pb virulence. The decrease in the amino acid content in the first week, compared to the control tissue, may be related to the use of tyrosine by Pb to synthesize melanin, a mechanism that increases its resistance to antifungal activities of macrophages [54]–[56] making it more resistant to the host immune system. The increase in the area after the second week of infection indicates the occurrence of a reaction of the body against Pb. Nevertheless it was observed that this increase is accompanied by a peak displacement in the region of 1595 cm^−1^, indicating a reduction in the hydroxylation process of phenylalanine.

The conversion of phenylalanine to tyrosine may have been affected by the reduction of the enzyme phenylalanine hydroxylase activity, an event that would explain such displacement detected until the eighth week of observation, when still had development of small numbers of CFUs.

It has been concluded that there was a directly proportional relation between the number of CFU, the number of granulomas and the physicochemical changes in the liver infected with Pb, that is: (a) periods of increased liver infection were the first two weeks, when there was also the largest number of granulomas and CFUs (b) periods of higher molecular and histopathological changes also corresponded to periods of increased infection. It was not possible to correlate the expression of gp43 with the physicochemical and morphological changes since the expression was similar in all periods.
